# O12 Pulseless electrical activity arrest in a young woman: could renal tubular acidosis due to Sjögren’s syndrome be the underlying cause?

**DOI:** 10.1093/rap/rkaa053.011

**Published:** 2020-11-03

**Authors:** Ali El Rashid, Myrto Cheila, Karen Douglas

**Affiliations:** The Dudley NHS Foundation Trust, Dudley, United Kingdom

## Abstract

**Case report - Introduction:**

Distal renal tubular acidosis (RTA) type 1 is a rare condition in adults, which is characterised by impaired distal urine acidification. It is caused by failure of alpha intercalated cells and presents with alkaline urine (pH.5.5), metabolic acidosis, hypokalaemia, osteomalacia, nephrocalcinosis and hypercalciuria. Autoimmune diseases, such as Sjögren’s Syndrome (SS) and Rheumatoid Arthritis are among the most common causes. RTA can be the presenting feature of the rheumatic disease. SS is typically characterised by impaired lacrimal and salivary gland function but has a broad spectrum of symptoms and disease severity, which includes renal involvement in up to 40%.

**Case report - Case description:**

We present the case of a 29-year-old female, referred to rheumatology by endocrinology following a recent emergency admission. The patient, who had woken with severe muscle paralysis of sudden onset, presented at A&E and imminently suffered a Pulseless Electrical Activity arrest. Urgent investigations identified profound hypokalaemia (serum potassium <1.5mmol/l), creatinine 120umol/l, a metabolic acidosis (bicarbonate 5.8 mmol/l), hypercalcemia (2.96 mmol/l) and hypermagnesemia (1.87mmol/l). She was successfully resuscitated and stabilised.

During her admission, under the care of endocrine and renal teams, a diagnosis of Distal RTA was reached. A CT scan showed medullary nephrocalcinosis. Immunology results: ANA 1:2560 (speckled pattern) and strongly positive Ro antibodies; polyclonal IgG of 19g/l, normal complement. Renal function returned to normal and she had negligible albuminuria. The causation of RTA was not certain. She was discharged well and on magnesium, potassium, and bicarbonate supplements. No renal biopsy was performed.

Past medical history included a pregnancy resulting in c-section (intrauterine growth retardation), six months previously. The baby was normal but post-partum the patient remained unwell with fatigue, nausea, and vomiting. She was diagnosed with postnatal depression. She had no other significant medical history but since the admission had suffered from a pneumonia and hearing loss due to recurrent otitis media.

As SS was considered as a possible cause of RTA, she was referred for a rheumatological opinion. She was fatigued but denied sicca symptomatology (Schirmer’s test was negative, and normal saliva production). She had a patch of alopecia, but no other classical features of SS. Salivary glands ultrasound were unremarkable. Her biochemistry was now normal except a low normal bicarbonate; inflammatory markers were within normal range.

**Case report - Discussion:**

This patient presents several challenges. She represents a common scenario of the balance between the high clinical suspicion and the failure to meet the ACR/EULAR diagnostic criteria for SS and the resultant management dilemma. She has no sicca symptoms, normal salivary gland US and hence biopsy was not sought. Anti-Ro/SSA and anti-La/SSB antibodies are detected on average 5 years before the appearance of an overt clinical phenotype of SS and thus serve also as predictive markers of the disease. In this patient with a strongly positive speckled ANA with anti RO along with RTA1(and renal microcalcification indicating long duration) without other explanation is almost certainly SS. The absence of renal biopsy in this patient also added to the challenge of clarification of her renal pathology.

Renal involvement  in SS is divided into: Overt glomerular disease which presents with haematuria, proteinuria or frank nephrotic syndrome; and Covert  tubular disease which manifests  with the metabolic abnormalities due to: proximal tubular injury; distal RTA or urinary concentrating defects.

The prevalence of overt (glomerular) renal disease in SS is 4.3-5%. The prevalence of covert disease is 10-42% (Proximal tubular injury 10-42%; Distal RTA 5-24%; urinary concentrating defect 17-28%).

Type1 RTA is confirmed by inappropriately alkaline urine (PH > 5.3) during systemic acidosis. The acidosis may occur spontaneously or be induced by an acid load test (NH4CL). Also, by stimulating intercalated cell acid secretion using a combination of fludrocortisone and furosemide.

In tubular disease, as in this patient, treatment to correct the metabolic abnormalities (with e.g. Bicarbonate) is normally required. However, as tubular interstitial nephritis (TIN) is the usual cause for renal tubular defects there is evidence that these patients respond to immunosuppression.

Monitoring of long-term effects, such as on bone metabolism, is required and thus patients warrant input from a multidisciplinary team for management.

**Case report - Key learning points:**

This case is a reminder of the considerable prevalence of renal involvement in Sjögren’s Syndrome. TIN is a typical manifestation of SS, and we should keep in mind the possible evolution to Distal RTA. Our routine clinical work up for SS includes a urine dip and we may focus on proteinuria and haematuria to monitor for glomerular disease and ignore the pH. We perhaps ought to routinely check calcium, magnesium bicarbonate in addition to renal function in patients with SS. A plain abdominal X Ray would detect nephrocalcinosis in chronic RTA. Beware nausea and vomiting may be a warning sign of tubular renal disease.

A renal biopsy is critical in the evaluation of renal disease in SS. Our renal team is reluctant to biopsy this patient. Thus, with the lack of tissue diagnosis, the patient not fulfilling ACR/EULAR criteria and the history of significant recent infections has made the introduction of immunosuppression difficult in this young woman.

